# Pharmacokinetic Study of rhIL-18BP and Its Effect on Radiation-Induced Cytokine Changes in Mouse Serum and Intestine

**DOI:** 10.3390/toxics11010035

**Published:** 2022-12-30

**Authors:** Wanchang Cui, Lisa Hull, Alex Zizzo, Li Wang, Bin Lin, Min Zhai, Mang Xiao

**Affiliations:** 1 Armed Forces Radiobiology Research Institute, Uniformed Services University of the Health Sciences, Bethesda, MD 20889, USA; 2 The Henry M. Jackson Foundation for the Advancement of Military Medicine, Inc., Bethesda, MD 20817, USA; 3 Department of Pharmacology and Molecular Therapeutics, Uniformed Services University of the Health Sciences, Bethesda, MD 20814, USA; 4 Department of Pathology, Uniformed Services University of the Health Sciences, Bethesda, MD 20814, USA

**Keywords:** ionizing radiation, IL-18, IL-18 binding protein, PK study, cytokine, serum, intestine

## Abstract

Administration of recombinant human IL-18 binding protein (rhIL-18BP), a natural antagonist of IL-18, significantly increased mouse survival after lethal doses of irradiation. To further understand the roles of IL-18BP in radiation mitigation, we studied the pharmacokinetic (PK) parameters of rhIL-18BP, and the serum and intestinal cytokine changes in CD2F1 mice treated with vehicle or rhIL-18BP after 9.0 Gy total body irradiation (TBI). For the PK study, non-compartmental pharmacokinetic analysis was performed using PKsolver. Serum and intestine specimens were collected to measure 44-cytokine levels. Principal component analysis showed a clear separation of the non-irradiated samples from the irradiated samples; and partial separation with or without rhIL-18BP treatment. Cytokine clusters that were significantly correlated in the serum or intestine, respectively were identified. On the individual cytokine levels, serum and intestinal cytokines that were significantly changed by irradiation and rhIL-18BP treatment were identified. Finally, cytokines that were significantly correlated between their serum and intestinal levels were identified. The current study established the PK parameters of rhIL-18BP in mice, identified significantly changed cytokines in mouse serum and intestine after radiation exposure and rhIL-18BP treatment. Current data provide critical insights into IL-18BP’s mechanism of action as a radiation mitigator.

## 1. Introduction

High dose ionizing radiation can cause serious health problems including hematopoietic (H-) and gastrointestinal (GI-) acute radiation syndrome (H-, and GI-ARS), which can ultimately lead to death [1,2]. IL-18, a member of the IL-1 family, was initially identified as “IFNγ-inducing factor” in the sera from P. acnes-primed and LPS-challenged mice [3]. IL-18 is a highly pleiotropic pro-inflammatory cytokine produced by various hematopoietic and nonhematopoietic cells, including endothelial cells (ECs), dendritic cells and macrophages and can propagate inflammation by promoting immune cell infiltration, leukocyte and lymphocyte activation and angiogenesis, and facilitates the transition from the innate to the adaptive immune response [4,5]. IL-18 binds to its receptor IL-18Rα to form a low affinity ligand chain. Further recruitment of IL-18Rβ can form a high affinity receptor complex to activate NF-κB and induce downstream inflammatory signaling [6,7]. IL-18 has been suggested to play important roles in radiation-induced tissue injury. It has been shown that IL-18 was up-regulated in mouse thymus, spleen, and bone marrow (BM) after total-body irradiation (TBI) [8]. IL-18 was significantly and persistently increased in mouse, minipig, and nonhuman primate (NHP) serum and/or urine in a radiation dose-dependent manner, and the highest levels of IL-18 were observed on day 3 after TBI [4,8,9].

IL-18 binding protein (IL-18BP) is a natural antagonist of IL-18 [10,11]. IL-18BP binds to the receptor-binding site of IL-18 with high affinity to block the IL-18 and IL-18 receptor complex formation and subsequently inhibits IL-18 activation [5]. There are four human isoforms (hIL-18BP a, b, c and d) and two murine isoforms (mIL-18BP c and d) of IL-18BP [12]. The hIL-18BPa and hIL-18BPc are the two functional isoforms of IL-18BP in human, while hIL-18BPb and hIL-18BPd are nonfunctional isoforms. Murine IL-18BPc and IL-18BPd are both functional isoforms in mouse. Among the isoforms, hIL-18BPa is the most effective isoform in inhibiting IL-18 in both human and mouse cells. Therefore, hIL-18BPa is widely used in preclinical and clinical studies. Recombinant human IL-18BP (or called Tadekinig Alfa) has been used for a phase II clinical trial in Europe to treat adult onset Still’s disease patients [13] and a phase III clinical trial with the experimental drug IL-18BP in patients carrying a mutation of the NOD-like receptor C4 (NLRC4) gene, characterized by severe, life threatening systemic inflammation associated with extremely high levels of IL-18 (http://www.ab2bio.com) [14] (accessed on 29 December 2022). More importantly, IL-18BP treatment has been shown to significantly increase mouse survival after lethal doses of radiation exposure [15].

Although the pharmacokinetic study of hIL-18BP has been performed on patients [16], there is no report of the pharmacokinetic study of hIL-18BP in rodents. The PK study in rodents provides important information about how long the drug persists in the rodent body so the appropriate dosing intervals can be achieved.

Cytokines are known to play important roles in radiation toxicity [17,18]. However, due to technical difficulties, usually only several cytokines can be measured each time. Current multiplexing arrays can increase the throughput while using small amounts of samples. In the current study, we investigated the effect of rhIL-18BP treatment on the radiation-induced cytokine changes in mouse serum and intestine at different time points using a 44-plex cytokine assay. The data will help to understand the mechanism of hIL-18BP’s radiation mitigator effect.

## 2. Materials and Methods

### 2.1. Ethics Statement

Animals were housed in an Association for Assessment and accreditation of Laboratory Animal Care (AAALAC)-approved facility at the Uniformed Services University of the Health Sciences (USUHS). All animal study procedures including housing, irradiation, survival study, and blood/tissue collection were reviewed and approved by the USUHS Institutional Animal Care and Use Committee (IACUC) and all experiments were performed in accordance with guidelines and regulations from the USUHS-IACUC and the USUHS Department of Laboratory Animal Resources (DLAR).

### 2.2. Mice and Animal Care

Twelve- to 14-week-old CD2F1 male mice (ENVIGO, Indianapolis, IN) were used for the current study. Mice were randomized for each experimental group. Animal rooms were maintained at 20–26 °C with 30–70% humidity on a 12 h light/dark cycle. Commercial rodent chow (Harlan Teklad Rodent Diet 8604) was available *ad libitum* as was acidified water (pH = 2.5–3.0) to control opportunistic infections.

### 2.3. PK Study

Recombinant human IL-18BPa (rhIL-18BP, Cat No. 119-BP; R&D Systems Inc., Minneapolis, MN, USA) was used in this study. rhIL-18BP is a water soluble small molecule. Saline was used as vehicle control in the animal studies when needed. 30 CD2F1 mice were administered a subcutaneous (s.c) injection of rhIL-18 BPa Fc Chimera protein, formulated in saline, at 2.0 mg/kg. The time of rhIL-18BP injection was considered as 0 h. Three mice (n = 3) were euthanized at each predetermined time point after drug injection (0.25, 0.5, 1, 2, 4, 8, 24, 48, 72, and 96 h) and the serum was collected immediately after euthanasia. Serum samples were used to measure the rhIL-18BP concentration using a Quantikine^®^ Human IL-18 BPa Immunoassay ELISA kit (Cat No. DBP180; R&D Systems Inc., Minneapolis, MN, USA) according to the manufacturer’s instructions. This ELISA kit is specific for human IL-18BP and has no significant cross-reactivity with mouse IL-18BP according to the manufactures’ test. Pharmacokinetic parameters were determined using a Microsoft Excel add-in “PKSolver” [19]. Noncompartmental analysis of serum data after extravascular input was used to determine the pharmacokinetic parameters.

### 2.4. Cytokine Study

#### 2.4.1. Irradiation and rhIL-18BP Treatment

Mice received TBI in a bilateral radiation field at AFRRI’s ^60^Co facility. The alanine/electron spin resonance (ESR) dosimetry system (American Society for Testing and Materials, Standard E 1607) was used to measure dose rates (to water) in the cores of acrylic mouse phantoms. The midline tissue dose to the mice was 9.0 Gy at a dose rate of 0.6 Gy/min. Control animals (0 Gy) were sham-irradiated, treated in the same manner as the irradiated animals except the ^60^Co source was not raised from the shielding water pool. The day of irradiation was considered day 0. rhIL-18BP was given subcutaneously (s.c) at d2 and d5 post TBI at a dose of 2 or 3 mg/kg.

#### 2.4.2. Tissue Collection and Cytokine Multi-Plex Assay

Mice were sacrificed at d3 and d7 after radiation exposure for tissue collection. The d3 mice received one dose of vehicle or rhIL-18BP treatment at d2; and the d7 mice received two doses of vehicle or rhIL-18BP treatment (d2 + d5). Control mice received neither radiation nor rhIL-18BP treatment. Serum and small intestine were collected at necropsy. Serum was mixed with equal volume of PBS and stored at −80 °C until cytokine assay. Intestinal samples were snap-frozen in liquid nitrogen and then stored at −80 °C until lysis. Intestinal samples were lyzed in RIPA buffer containing proteinase inhibitor, and adjusted to the same concentration of 3 mg/mL for cytokine assay.

Prepared serum and intestinal lysate were analyzed to determine the concentrations of 44 cytokines using the Mouse Cytokine/Chemokine 44-Plex Discovery Assay^®^ Array (MD44) (Eve Technologies, Calgary, Alberta, Canada). These targets include Eotaxin, Erythropoietin, 6Ckine, Fractalkine, G-CSF, GM-CSF, IFNB1, IFNγ, IL-1α, IL-1β, IL-2, IL-3, IL-4, IL-5, IL-6, IL-7, IL-9, IL-10, IL-11, IL-12p40, IL-12p70, IL-13, IL-15, IL-16, IL-17, IL-20, IP-10, KC, LIF, MCP-1, MCP-5, M-CSF, MDC, MIG, MIP-1α, MIP-1β, MIP-2, MIP-3α, MIP-3B, RANTES, TARC, TIMP-1, TNFα, and VEGF-A.

### 2.5. Statistical Analysis

Principal component analysis (PCA), correlation, and differences between groups were analyzed using Graphpad Prism Software version 9.4.1 (GraphPad Software, San Diego, CA, USA). Group differences were analyzed using one-way ANOVA, with Tukey’s multiple comparison tests between groups. *p* < 0.05 is considered significantly different. N = 5–6 mice/group.

## 3. Results

### 3.1. Pharmacokinetic Analysis of rhIL-18BP in CD2F1 Mice

**Figure 1:**
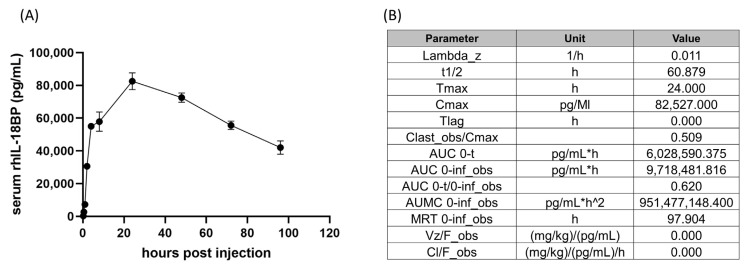
The PK study of rhIL-18BP in CD2F1 mice. rhIL-18BP was injected s.c into CD2F1 mice. Serum samples from 0.25, 0.5, 1, 2, 4, 8, 24, 48, 72, and 96 h after injection were used for rhIL-18BP quantification using ELISA. Pharmacokinetic parameters were determined using a Microsoft Excel add-in “PKSolver”. (**A**) Serum concentrations of rhIL-18BP at different time points after injection. (**B**) PK parameters generated from the PKSolver using data from (**A**).

CD2F1 male mice were injected with 2.0 mg/kg rhIL-18BP s.c. The serum concentrations of rhIL-18BP over the time course of 0.25 to 96 h after injection were plotted in Figure 1A. Serum rhIL-18BP showed an initial steep increase from 0.25 h to 4 h after injection, reaching ng/mL levels. Thereafter, serum rhIL-18BP still increased until 24 h when it reached its peak with a much slower rate, and then started to decrease slowly after 24 h all the way to 96 h after injection. Important pharmacokinetic parameters such as the half-life (t1/2), the time of maximal concentration (Tmax), the maximal concentration (Cmax), the area under the curve (AUC) were listed in Figure 1B. Tmax was 24 h and half-life was 60.9 h after injection for rhIL-18BP in mice.

### 3.2. Principal Component Analysis of Mouse Serum Cytokines after Irradiation and IL-18BP Treatment

**Figure 2:**
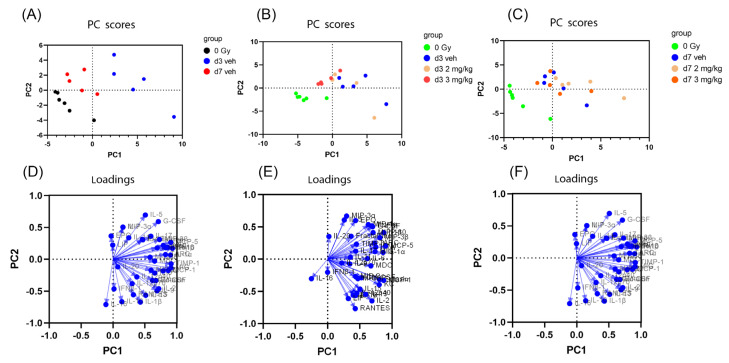
PCA analysis of serum cytokines in CD2F1 mice with irradiation and/or rhIL-18BP treatment. (**A**) PC score plot of non-irradiated mice with d3 and d7 irradiated vehicle treatment mice. (**B**) PC score plot of non-irradiated mice, and d3 irradiated mice (treated with vehicle or 2 or 3 mg/kg rhIL-18BP). (**C**) PC score plot of non-irradiated mice, and d7 irradiated mice (treated with vehicle or 2 or 3 mg/kg rhIL-18BP). (**D**), loading score plot of (**A**). (**E**), loading score plot of (**B**). (**F**), loading score plot of (**C**).

Among the 44 cytokine targets, 3 were below the detection level in all serum samples (IL-3, IL-4 and VEGF), and 1 cytokine was above the detection level in all serum samples (6Ckine/Exodus2). Therefore, these 4 cytokines were excluded for analysis in the serum samples. Because of the large number of cytokines (around 40 cytokines), we used PCA to examine the influence of all the cytokines simultaneously in detecting patterns of cytokine variation between the groups. To simplify the results, we performed PCA analysis in (A) 3 groups: 0 Gy, d3 TBI + vehicle, and d7 TBI + vehicle groups (no rhIL-18BP treatment), (B) 4 groups: 0 Gy, d3 TBI + vehicle, d3 TBI + 2 mg/kg rhIL-18BP, and d3 TBI + 3 mg/kg rhIL-18BP, and (C) 4 groups: 0 Gy, d7 TBI + vehicle, d7 TBI + 2 mg/kg rhIL-18BP, and d7 TBI + 3 mg/kg rhIL-18BP (Figure 2). There were clear separations between 0 Gy, d3 and d7 TBI + vehicle groups as shown in Figure 2A. At day 3, there was clear separation between the 0 Gy samples compared to any d3 post-TBI groups; d3 TBI + vehicle group could be separated from the d3 TBI + 3 mg/kg rhIL-18BP group but not the d3 TBI + 2 mg/kg rhIL-18BP group. At day 7, there was clear separation between the 0 Gy group compared to any post-irradiated samples; however, there was no clear separation between any post-irradiated groups. Loadings are the coefficients of the linear combination of the original variables from which the principal components are constructed. A variable with higher loading value has a bigger effect than other variables with smaller loading values. The loading values were shown in Figure 2D–F corresponding with each PCA analysis in Figure 2A–C, respectively. The loading value plots suggested that no single or few variables (i.e., cytokines) had prominent effect in the principal component analysis. Therefore, multiple variables (i.e., cytokines) were needed to explain the difference between the groups.

### 3.3. Significantly Correlated Serum Cytokines

**Figure 3:**
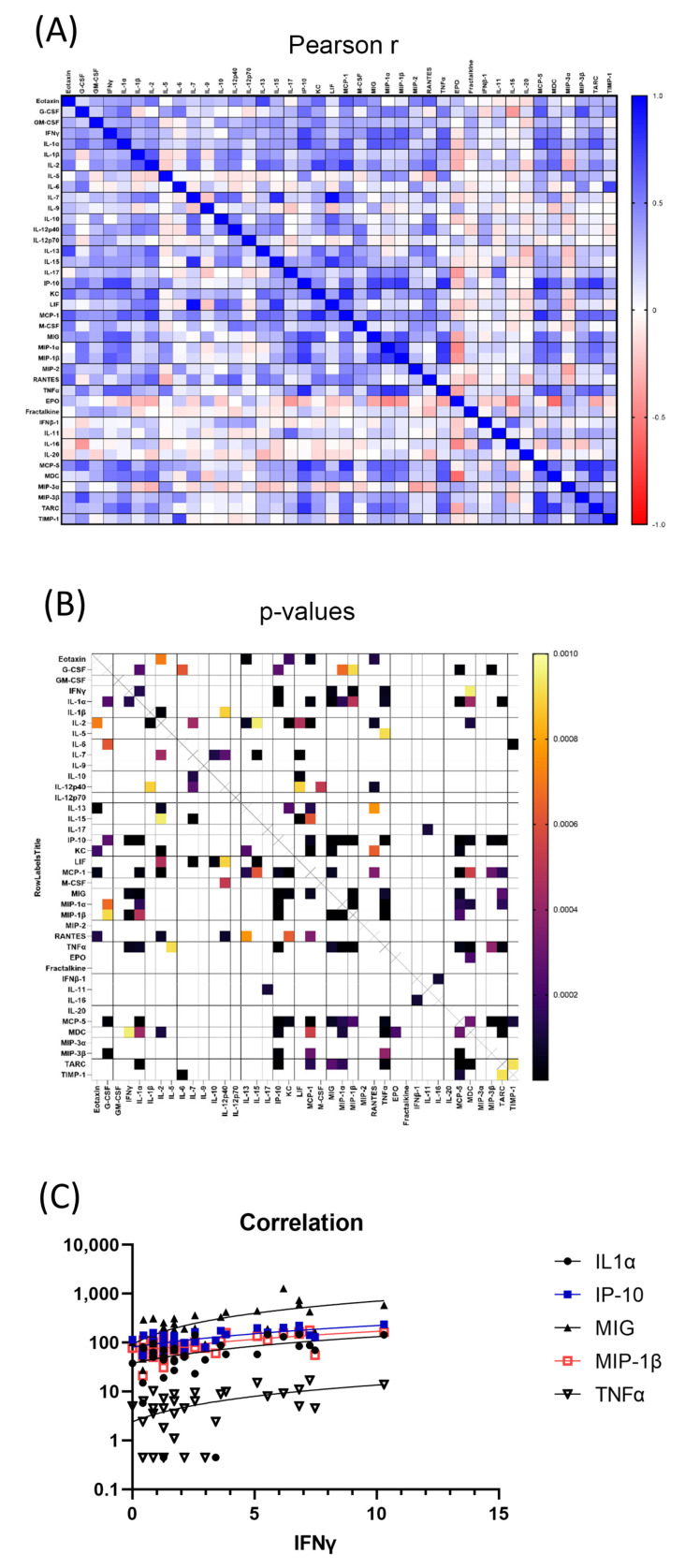
Correlation matrix of serum cytokines. All 40 serum cytokines were compared to each other using Pearson correlation. (**A**) Pearson *r* value heatmap of all serum cytokine combinations. Legend was shown on the right. *r* values closer to 1 or −1 means positively or negatively correlated. *R* values closer to 0 means no correlation. (**B**) *p* value heatmap of all serum cytokine combinations. Legend was shown on the right. Only *p* values < 0.001 were shown to simplify the view. (**C**) Serum cytokines that were significantly correlated with serum IFNγ. All their *p* values were smaller than 0.001. All cytokines’ concentration unit was pg/mL.

Cytokines that are significantly correlated potentially affect each other or work in the same pathway. The correlation between the 40 serum cytokines in all the 36 mouse samples were computed to find significantly related pairs. The correlation matrix showing the Pearson *r* values and *p* values were shown in Figure 3A,B for the serum cytokines. The correlation matrix showed that there were many serum cytokine clusters significantly correlated. For example, IFNγ was significantly correlated with IL-1α, IP-10, MIG, MIP-1β and TNFα (Figure 3C), thus forming a cluster of serum cytokines that were significantly correlated; while GM-CSF was not significantly correlated with any cytokine.

### 3.4. Individual Mouse Serum Cytokine Changes after 9.0 Gy TBI

Each serum cytokine levels in the mice irradiated with 9.0 Gy TBI at d3 and d7 with vehicle treatment were compared to that of the non-irradiated controls (Table 1). At d3 after TBI, 16 cytokines were significantly elevated compared to the 0 Gy controls, including MCP-1, MDC, MIP-3β, IL-17, MIG, MIP-1α, TARC, IL-5, IL-1α, TNFα, TIMP-1, MCP-5, G-CSF, IFNγ, IP-10, MIP-1β, and IL-16. At d7 after 9.0 Gy TBI, 6 cytokines were significantly elevated, including MIP-3β, IL-5, MCP-5, G-CSF, MIP-3α, and EPO; and one cytokine was significantly decreased, IL-16. Among the significantly changed serum cytokines, only 4 cytokines were significantly elevated at both d3 and d7 post irradiation, including MIP-3β, IL-5, MCP-5, and G-CSF.

### 3.5. Serum Cytokines That Were Significantly Changed by rhIL-18BP Treatment after 9.0 Gy TBI

**Figure 4:**
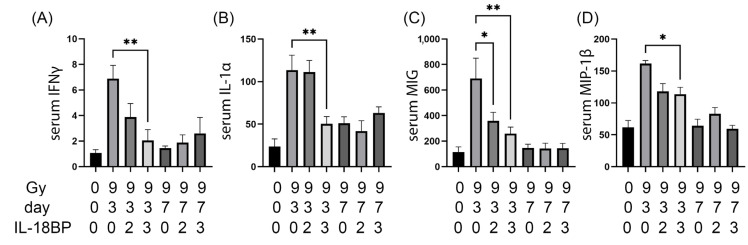
Serum cytokines that were significantly changed by rhIL-18BP treatment after irradiation. Radiation dose, time points after irradiation, and rhIL-18BP dose (mg/kg) were shown below the graphs. (**A**) serum IFNγ levels, (**B**) serum IL-1α levels, (**C**) serum MIG levels, and (**D**) serum MIP-1β levels. All cytokines’ concentration unit was pg/mL. 0 Gy samples were not included for statistical analysis to simplify the view. *, *p* < 0.05; **, *p* < 0.01.

rhIL-18BP treatment significantly decreased 4 out of the 44 serum cytokines at d3 after 9.0 Gy TBI, including IFNγ, IL-1α, MIG and MIP-1β (Figure 4). The effect of rhIL-18BP treatment was dose- and time-dependent. 3 mg/kg rhIL-18BP treatment significantly decreased the serum levels of IFNγ, IL-1α, MIP-1β at d3 only (Figure 4 A,B,D), while both 2 and 3 mg/kg rhIL-18BP treatment significantly decreased serum MIG levels at d3 post TBI (Figure 4C).

### 3.6. Principal Component Analysis of Mouse Intestinal Cytokines after Irradiation and rhIL-18BP Treatment

**Figure 5:**
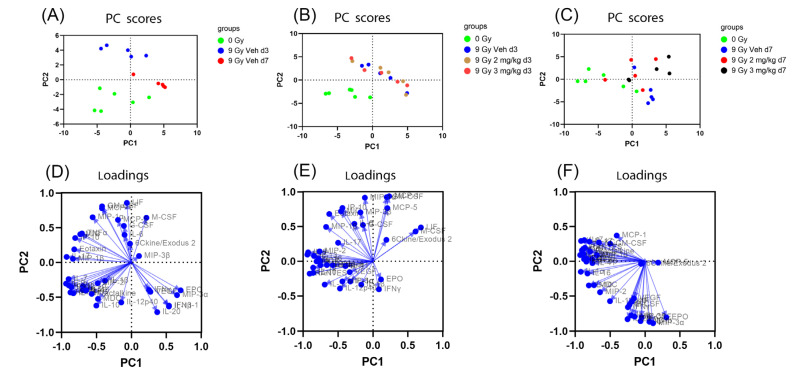
PCA analysis of intestinal cytokines in CD2F1 mice with irradiation and/or rhIL-18BP treatment. (**A**) PC score plot of non-irradiated mice with d3 and d7 irradiated vehicle treatment mice. (**B**) PC score plot of non-irradiated mice, and d3 irradiated mice (treated with vehicle or 2 or 3 mg/kg rhIL-18BP). (**C**) PC score plot of non-irradiated mice, and d7 irradiated mice (treated with vehicle or 2 or 3 mg/kg rhIL-18BP). (**D**), loading score plot of (**A**). (**E**), loading score plot of (**B**). (**F**), loading score plot of (**C**).

Besides two intestinal cytokines below detection in all samples (IL-3 and IL-12p70), all 42 cytokines were above the detection limit of the assay in the intestinal samples. Similarly, we used PCA to examine the influence of all the cytokines simultaneously in detecting patterns of cytokine variation between the groups (Figure 5). To simplify the results, we performed PCA analysis in (A) 3 groups: 0 Gy, d3 TBI + vehicle, and d7 TBI + vehicle groups (no rhIL-18BP treatment), (B) 4 groups: 0 Gy, d3 TBI + vehicle, d3 TBI + 2 mg/kg rhIL-18BP, and d3 TBI + 3 mg/kg rhIL-18BP, and (C) 4 groups: 0 Gy, d7 TBI + vehicle, d7 TBI + 2 mg/kg rhIL-18BP, and d7 TBI + 3 mg/kg rhIL-18BP (Figure 5). There was clear separation between 0 Gy, d3 TBI + vehicle and d7 TBI + vehicle groups as shown in Figure 5A. At day 3, there was clear separation between the 0 Gy samples compared to any d3 irradiated groups; however, there was no separation within any d3 irradiated groups (Figure 5B). At day 7, there was no clear separation between any groups (Figure 5C). The loading values were shown in Figure 5D–F corresponding with each PCA analysis in Figure 5A–C, respectively. Similar to the serum data, the loading value plots suggested that no single or few variables (i.e., cytokines) had prominent effect in the principal component analysis. Therefore, multiple variables (i.e., cytokines) were needed to explain the difference between the groups.

### 3.7. Significantly Correlated Mouse Intestinal Cytokines

**Figure 6:**
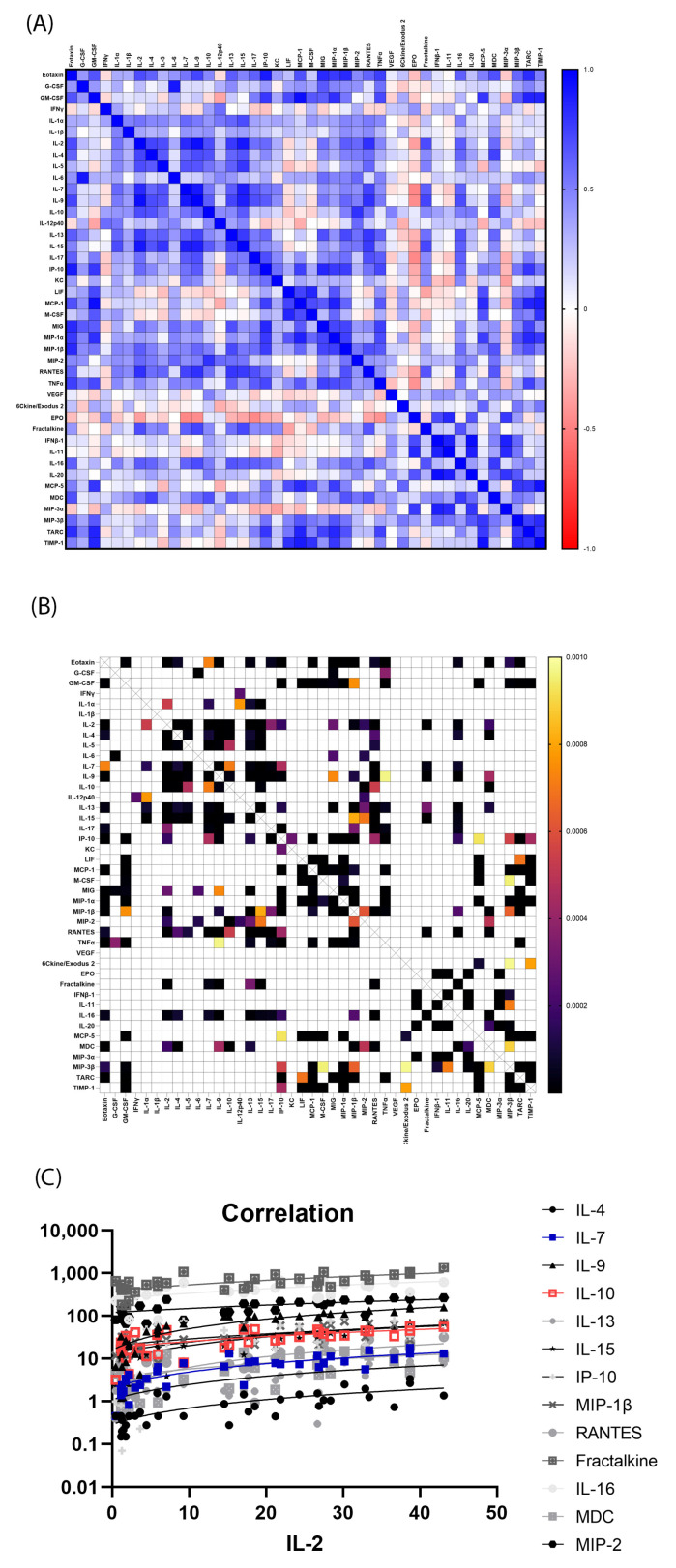
Correlation matrix of intestinal cytokines. All 42 intestinal cytokines were compared to each other using Pearson correlation. (**A**) Pearson *r* value heatmap of all intestinal cytokine combinations. Legend was shown on the right. *r* values closer to 1 or −1 means positively or negatively correlated. *R* values closer to 0 means no correlation. (**B**) *p* value heatmap of all intestinal cytokine combinations. Legend was shown on the right. Only *p* values < 0.001 were shown to simplify the view. (**C**) Intestinal cytokines that were significantly correlated with intestinal IL-2. All their *p* values were smaller than 0.001. All cytokines’ concentration unit was pg/mL.

The correlation between the 42 intestinal cytokines in all the 36 mouse samples were computed to find significantly related pairs. The correlation matrix showing the Pearson *r* values and *p* values were shown in Figure 6A,B for the intestinal cytokines. The correlation matrix showed there were many cytokine clusters that were significantly correlated. For example, intestinal IL-2 was significantly correlated with intestinal IL-4, IL-7, IL-9, IL-10, IL-13, IL-15, IP-10, MIP-1β, RANTES, Fractalkine, IL-16, MDC and MIP-2 (Figure 6C), thus forming a cluster of intestinal cytokines that were significantly correlated; while intestinal IFNγ was only significantly correlated with one intestinal cytokine.

### 3.8. Individual Mouse Intestinal Cytokine Changes after 9.0 Gy TBI

The individual intestinal cytokine levels in the mice irradiated with 9.0 Gy TBI at d3 and d7 vehicle groups were compared to that of the non-irradiated mice (Table 2). At d3 post TBI, 5 intestinal cytokines were significantly elevated compared to the 0 Gy controls, including M-CSF, TIMP-1, GM-CSF, LIF, and MCP-1; one cytokine RANTES was significantly decreased compared to the 0 Gy controls. At d7 post TBI, 8 intestinal cytokines were significantly decreased compared to the 0 Gy controls, including RANTES, IL-2, IL-4, IL-5, IL-9, IL-13, MIP-1β, and MDC. Among the significantly changed intestinal cytokines, only RANTES showed persistent change at d3 and d7 after TBI.

### 3.9. Intestinal Cytokines Significantly Changed by rhIL-18BP Treatment after 9.0 Gy TBI

**Figure 7:**
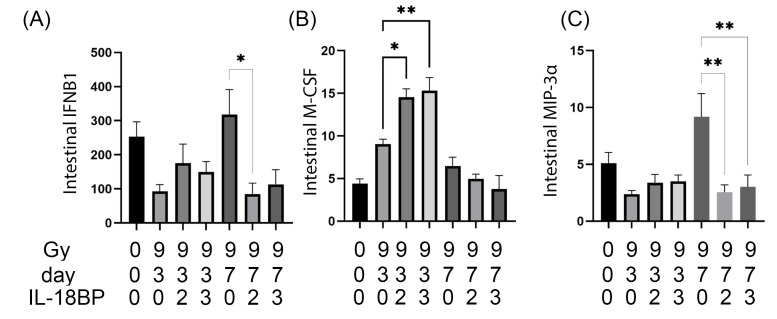
Intestinal cytokines that were significantly changed by rhIL-18BP treatment after irradiation. Radiation dose, time points after irradiation, and IL-18BP dose (mg/kg) were shown below the graphs. (**A**) intestinal IFNB1 levels, (**B**) intestinal M-CSF levels, (**C**) intestinal MIP-3α levels. All cytokines’ concentration unit was pg/mL. 0 Gy samples were not included for statistical analysis to simplify the view. *, *p* < 0.05; **, *p* < 0.01.

rhIL-18BP treatment significantly decreased 3 out of the 44 intestinal cytokines at either d3 or d7 post 9.0 Gy TBI, including IFNB1, M-CSF, and MIP-3α (Figure 7). rhIL-18BP treatment significantly decreased levels of IFNB1 and MIP-3α at d7 only (Figure 7A,C), but significantly increased the intestinal levels of M-CSF at d3 only (Figure 7B). Both 2 and 3 mg/kg rhIL-18BP treatment had significant effect on intestinal M-CSF and MIP-3α levels (Figure 7B,C), while only 2 mg/kg rhIL-18BP treatment had significant effect on IFNB1 levels post TBI (Figure 7A).

### 3.10. Significantly Correlated Intestinal and Serum Cytokines

**Figure 8:**
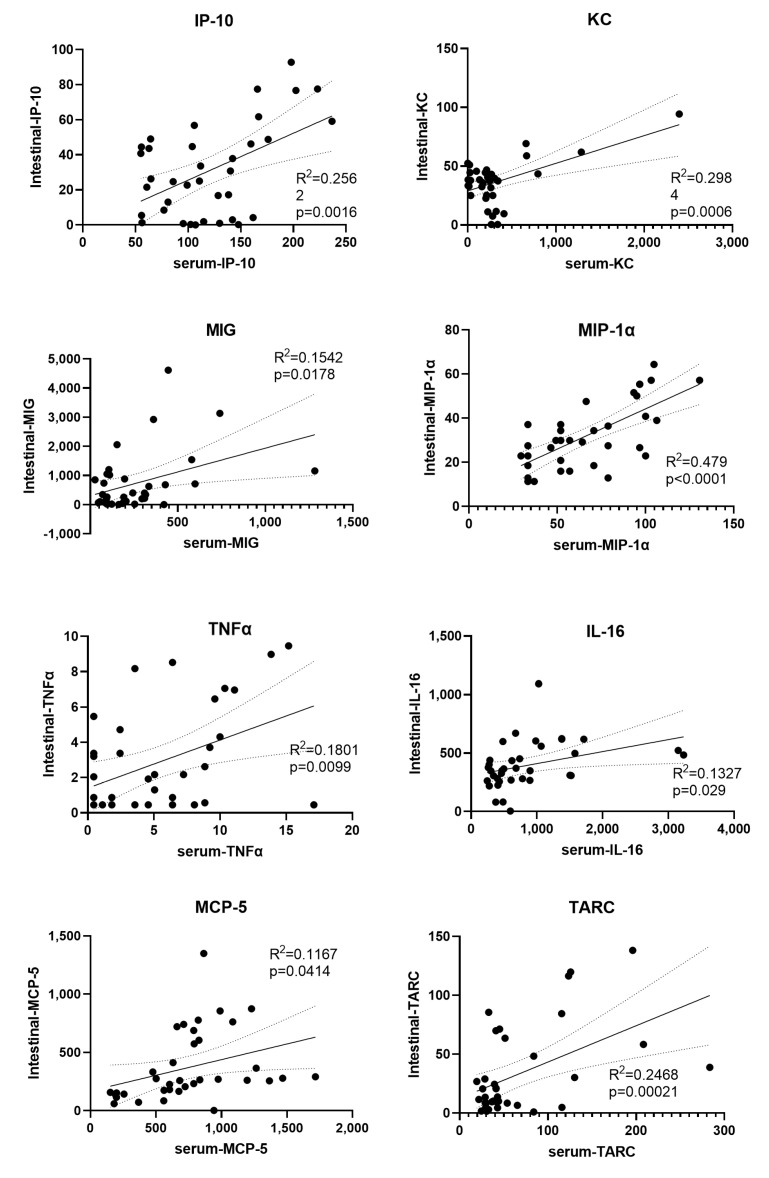
Significantly correlated cytokines between serum and intestine. Pearson’s correlation analysis was performed. Each dot represented one mouse. Pearson’s correlation coefficient (*R*^2^) and *p* values for each cytokine were shown on each graph. Black line was the linear regression line and dotted lines represented the 95% confidence intervals of the linear regression line.

Significant correlation of a cytokine in the intestine and serum may suggest the source of this circulating cytokine. The serum and intestinal levels of each cytokine were computed and there were 8 cytokines significantly correlated between its serum and intestinal levels (Figure 8), including IP-10, KC, MIG, MIP-1α, TNFα, IL-16, MCP-5 and TARC.

## 4. Discussion

### 4.1. Pharmacokinetics of hIL-18BP in Human and Mice

Pharmacokinetics study of rhIL-18BP has been performed on healthy human volunteers [16]. rhIL-18BP showed a dose-dependent pharmacokinetic profile, with peak serum concentrations of 12–20 h after single injection in patients, similar to 24 h as shown in our current study (Figure 1). rhIL-18BP’s serum half-life after single injection in healthy human volunteers were 33–40 h, which is shorter than the 60 h in our current study (Figure 1), suggesting a faster elimination in humans than mice. The difference may be explained by the fact that the current study used a Fc chimera form of the rhIL-18BP while the human study used rhIL-18BP itself. The Fc fused proteins generally have a longer half-life by both increasing the protein size and higher recycling process [20]. Our current data suggest that repeated rhIL-18BP injection of 72 h intervals helps to maintain the steady state of its serum levels in mice.

### 4.2. Radiation-Induced Cytokine Storms

Although radiation is known to cause cytokine storm (systemic inflammatory reaction involving elevated levels of circulating cytokines and the excessive activation of the immune system), it is mostly reported in fractionated radiotherapy [21] or local irradiation models such as thoracic irradiation [22,23]. There is great interest to study serum or tissue cytokine changes in mice exposed to TBI and countermeasure treatment [24,25]. In our current report, using a multiplex screen for 44 cytokines/chemokines, we studied the serum and intestinal cytokine changes after total body radiation at different time-points in mice. Our data support the idea of radiation-induced cytokine storm, as the serum and intestinal PCAs showed clear separation between the non-irradiated and irradiated samples at d3 and d7 based on the cytokine profiles (Figure 2 and Figure 5). No single or few cytokines could account for the difference between the non-irradiated and irradiated samples, suggesting that multiple or combined cytokine changes underlie the radiation-induced effect. The current data also suggest that blocking single or few significantly changed cytokines may have limited effect on mitigating radiation-induced toxicity because other cytokines also play important roles. Multi-cytokine inhibitors may yield better survival benefits than single inhibitor after TBI.

Monotherapy targeting one cytokine may be effective and associated with fewer side effects compared to combination therapy targeting two or more cytokines. For example, anti-TNFα is an effective drug for rheumatoid arthritis and Crohn’s diseases [26,27]. However, combination of several cytokine inhibitors may be more effective. For example, combination of anti-IL1 and TNFα provides synergistic effect on the treatment of rheumatoid arthritis [28]. Even in the case of a Macrophage Activation Syndrome (MAS), where IL-18 has a causal effect [29], the patient received combined IL-1β and IL-18 blockade for 11 months [14]. In radiation toxicity, at least based on our current work, not a single or few cytokines can explain the radiation toxicity. Therefore, as we suggested, blocking or regulating one key cytokine may be effective, as shown in our publication [15]. Combination of rhIL-18BP with other cytokine inhibitors can be used to keep the target cytokines in a healthy or balanced (normal) levels. This may have a better effect on increasing survival or decreasing toxicity.

Our data also identified many cytokine clusters that may play important roles in H-ARS and GI-ARS. For examples, clusters of cytokines including serum IFN-γ, IL-1α, IP-10, MIG, MIP-1β and TNFα were significantly correlated (Figure 3C). IFN-γ are known to orchestrate the traffic of specific immune cells to sites of inflammation through up-regulating expression of many specific cytokines, such as IP-10, MIG and MIP-1β [30]. IP-10 and MIG are particularly related with IFNγ as IP-10′s full name is IFN-γ inducible protein 10 and MIG’s full name is monokine induced by IFNγ. However, in the intestine, there was almost no cytokine significantly correlated with IFN-γ, suggesting that the IFNγ cytokine cluster may play important roles in H-ARS, but not GI-ARS. On the other hand, IL-2 cytokine cluster may play important roles in both H-ARS and GI-ARS.

On the individual cytokine levels, our data also showed there were specific and temporal cytokine changes after radiation exposure in both serum and intestine (Table 1 and Table 2). On day 3, all or most of the significantly changed cytokines were increased compared to baseline levels in both serum and intestine; while that is still the case in serum, all the significantly changed cytokine in the intestine were decreased compared to the baseline levels at day 7. Most significantly changed cytokines are pro-inflammatory cytokines (such as MCP-1, TNFα, IFNγ etc), therefore, it would make sense to inhibit these cytokines to decrease radiation-induced toxicity. G-CSF was shown to be highly elevated in the serum at both d3 and d7 (Table 1), which is in agreement with reported literature showing that radiation increased G-CSF levels after TBI [31]. However, blocking G-CSF exacerbated the deleterious effect of radiation [31] and G-CSF is a FDA-approved mitigator for H-ARS [32]. Therefore, careful consideration of modulating the identified cytokines is needed to achieve the desired effect.

RANTES, also called CCL5, was not significantly changed in serum but significantly decreased in the intestine at d3 and d7 after TBI (Table 1 and Table 2). RANTES is a pluripotent cytokine and plays important roles in inflammation; it is produced by a variety of cells, such as platelets, macrophages, eosinophils, fibroblasts, endothelium, epithelia and endometrial cells [33]. A recent study reported that injection of RANTES (as an endothelial cell-secreted hematopoietic growth factor) after 5 Gy TBI promoted hematopoietic cells and increased survival of C57BL/6 mice [34]. It is currently unclear why the RANTES levels went down in the intestine after radiation exposure. It is possible that RANTES producing cells were killed by lethal dose radiation (9.0 Gy) at early time points after radiation exposure. Radiation-induced chronic intestinal inflammation (radiation enteritis) usually manifest several months to years after radiation exposure. RANTES level may be elevated at later time points. Future studies are needed to clarify the changes of RANTES levels after radiation exposure.

### 4.3. The Effect of rhIL-18BP on the Radiation-Induced Cytokine Changes

On the d3 serum samples, there was a clear separation between the irradiated vehicle group and the 3 mg/kg hIL-18BP group (Figure 2B). There was no clear separation between the rhIL-18BP groups at other dose or time points, both in serum and intestine. This result may be in agreement with our published results showing that hIL-18BP increased survival after TBI but to a limited extent [15]. Combination of rhIL-18BP with other cytokine inhibitors may yield better effect on increasing survival or decreasing toxicity. On the individual cytokine levels, we identified rhIL-18BP treatment significantly decreased the levels of IFNγ, IL-1α, MIG and MIP-1β in the serum, which is in agreement with our published data showing that hIL-18BP decreased the IFNγ levels in the bone marrow after TBI [15], supporting IL-18BP’s mechanism of action is to inhibit the IFNγ pathway in H-ARS.

In our published data, we showed that treatment with rhIL-18BP significantly decreased free IL-18 levels at d1 or d3; and increased complete blood counts after radiation exposure (including white blood cells, neutrophils, and platelets) at d7 after radiation exposures, through inhibiting IL-18 induced IFNγ production in bone marrow [15]. Similar to radiation-induced bone marrow aplasia, elevated or prolonged IFNγ has been suggested to cause bone marrow failure in aplastic anemia, HIV and graft-versus-host diseases, by modifying cytokine responses and expression of genes involved in hematopoietic stem cell proliferation [35,36]. Our data suggest that radiation increases the levels of IL-18, thus causing elevated levels of IFNγ in bone marrow. As a negative modulator of hematopoietic stem cell self-renewal, elevated IFNγ decreases hematopoietic blood cells proliferation. IL-18BP treatment mitigates the effect of IL-18 and IFNγ on the hematopoietic blood cells, thus increasing survival.

### 4.4. Correlated Cytokines between the Serum and Intestine

We identified 8 cytokines that were significantly correlated between its serum and intestinal levels (Figure 8), including IP-10, KC, MIG, MIP-1α, TNFα, IL-16, MCP-5 and TARC. The significant correlation between these cytokines suggest that at least part of these serum cytokines may come from the intestine.

## 5. Conclusions

In summary, our data established the PK parameters of rhIL-18BP in mice and studied its effect on the serum and intestinal cytokines after 9.0 Gy TBI. Our data support the idea of radiation-induced cytokine storm and that IL-18BP mitigates H-ARS by inhibiting IFNγ signaling.

## Figures and Tables

**Table 1:** Individual serum cytokine changes after 9.0 Gy TBI in CD2F1 male mice. Serum cytokine levels at d3 and d7 after irradiation with vehicle treatment were compared to the 0 Gy controls. All cytokines’ concentration unit was pg/mL. Mean ± standard error of mean (SEM) were shown for each cytokine. Red color represented significantly elevated cytokines and green color represented significantly decreased cytokines compared to 0 Gy controls. n.d, non-detectable. a.d, above detection. n.s, non significant. N = 5–6 mice/group. *p* < 0.05 is considered significantly changed. Mouse serum cytokine changes after 9 GyTBI.

	Cytokines	0 Gy Concentration	9 Gy d3 veh Concentration	*p* Value d3 veh vs. 0 Gy	9 Gy d7 veh Concentration	*p* Value 9 Gy d7 veh vs. 0 Gy
1	MCP-1	13.31 ± 1.97	495.87 ± 194.8	*p* < 0.05	138.79 ± 32.75	n.s
2	MDC	325.67 ± 42.59	513.38 ± 72.71	*p* < 0.05	187.58 ± 22.82	n.s
3	MIP-3β	178.38 ± 24.48	320.74 ± 11.59	*p* < 0.01	283.41 ± 34.49	*p* < 0.05
4	IL-17	3.12 ± 0.37	8.16 ± 1.56	*p* < 0.01	3.33 ± 0.89	n.s
5	MIG	112.85 ± 42.23	690.56 ± 159.29	*p* < 0.01	146.93 ± 29.25	n.s
6	MIP-1α	46.67 ± 7.3	95.7 ± 10.46	*p* < 0.01	42.61 ± 4.72	n.s
7	TARC	29.73 ± 3.86	132.74 ± 31.44	*p* < 0.01	43.26 ± 6.11	n.s
8	IL-5	4.75 ± 1.59	23.37 ± 3.52	*p* < 0.001	18.5 ± 0.94	*p* < 0.01
9	IL-1α	23.77 ± 9.04	113.51 ± 17.61	*p* < 0.001	51.19 ± 7.54	n.s
10	TNFα	2.16 ± 0.83	12.25 ± 1.44	*p* < 0.001	2.67 ± 1.56	n.s
11	TIMP-1	1627.59 ± 330.01	4850.59 ± 707.44	*p* < 0.001	1611.3 ± 190.79	n.s
12	MCP-5	196.13 ± 14.01	1112.96 ± 140.57	*p* < 0.0001	672.18 ± 64	*p* < 0.01
13	G-CSF	378.82 ± 213.87	12,031.13 ± 1317.17	*p* < 0.0001	7820.85 ± 1429.67	*p* < 0.001
14	IFNγ	1.07 ± 0.26	6.88 ± 1.04	*p* < 0.0001	1.45 ± 0.17	n.s
15	IP-10	66.32 ± 6.74	190.45 ± 19.34	*p* < 0.0001	104.05 ± 10	n.s
16	MIP-1β	61.82 ± 10.59	161.69 ± 4.78	*p* < 0.0001	64.12 ± 10.51	n.s
17	IL-16	1957.48 ± 396.85	935.04 ± 162.28	n.s	429.42 ± 86.09	*p* < 0.01
18	MIP-3α	80.5 ± 23.53	206.4 ± 44.29	n.s	343.82 ± 55.68	*p* < 0.01
19	EPO	246.74 ± 32.09	340.36 ± 30.09	n.s	791.16 ± 110.13	*p* < 0.001
20	Eotaxin	826.05 ± 160.13	1472.6 ± 361.65	n.s	1357.4 ± 222.35	n.s
21	GM-CSF	3.96 ± 1.67	6.76 ± 3.06	n.s	2.24 ± 1.09	n.s
22	IL-1β	3.79 ± 0.45	3.81 ± 0.53	n.s	2.85 ± 0.69	n.s
23	IL-2	3.25 ± 0.8	6.16 ± 2.31	n.s	2.04 ± 0.95	n.s
24	IL-3	n.d	n.d	n.s	n.d	n.s
25	IL-4	n.d	n.d	n.s	n.d	n.s
26	IL-6	6.42 ± 3.28	26.44 ± 12.7	n.s	18.32 ± 7.34	n.s
27	IL-7	0.51 ± 0.03	1.1 ± 0.64	n.s	0.45 ± 0	n.s
28	IL-9	6.27 ± 1.33	11.23 ± 3.97	n.s	5.48 ± 2.08	n.s
29	IL-10	5.96 ± 1.9	4.3 ± 1.28	n.s	2.63 ± 0.84	n.s
30	IL-12p40	1.52 ± 1.06	2.88 ± 1.18	n.s	2.3 ± 1.26	n.s
31	IL-12p70	2.47 ± 2.02	2.38 ± 1.09	n.s	0.82 ± 0.37	n.s
32	IL-13	43.27 ± 5.81	48.95 ± 10.74	n.s	48.22 ± 6.14	n.s
33	IL-15	3.84 ± 1.58	27.39 ± 13.8	n.s	2.54 ± 0.27	n.s
34	KC	12.45 ± 4.9	812.75 ± 408.45	n.s	241.88 ± 62.98	n.s
35	LIF	0.73 ± 0.28	0.82 ± 0.31	n.s	0.45 ± 0	n.s
36	M-CSF	7.16 ± 1.83	11.87 ± 1.41	n.s	7.77 ± 1.55	n.s
37	MIP-2	141.3 ± 2.91	147.92 ± 2.69	n.s	138.06 ± 1.44	n.s
38	RANTES	19.32 ± 2.81	21.6 ± 7.15	n.s	22.69 ± 4.77	n.s
39	VEGF	n.d	0.86 ± 0.32	n.s	n.d	n.s
40	6Ckine/Exodus 2	a.d	a.d	n.s	a.d	n.s
41	Fractalkine	201.36 ± 19.64	370.76 ± 119.56	n.s	250 ± 43.94	n.s
42	IFNβ-1	138.23 ± 59.63	75.54 ± 9.65	n.s	48.13 ± 1.62	n.s
43	IL-11	25.75 ± 3.47	39.79 ± 11.55	n.s	28.57 ± 19.57	n.s
44	IL-20	78.37 ± 18.03	81.86 ± 11.61	n.s	67.13 ± 11.64	n.s

**Table 2:** Intestinal cytokine changes after 9.0 Gy TBI in CD2F1 mice. Intestinal cytokine levels at d3 and d7 after irradiation with vehicle treatment were compared to the 0 Gy controls. All cytokines’ concentration unit was pg/mL. Mean ± standard error of mean (SEM) were shown for each cytokine. Red color represented significantly elevated cytokines and green color represented significantly decreased cytokines. n.d, non-detectable. n.s, non significant. N = 5–6 mice/group. *p* < 0.05 is considered significantly changed. Mouse intestinal cytokine changes after 9 GyTBI.

	Cytokines	0 Gy Concentrations	9 Gy d3 veh Concentrations	*p* Value d3 veh vs. 0 Gy	9 Gy d7 veh Concentrations	*p* Value 9 Gy d7 veh vs. 0 Gy
1	M-CSF	4.39 ± 0.56	9.02 ± 0.6	*p* < 0.05	6.48 ± 1.03	n.s
2	TIMP-1	176.41 ± 20.02	610.07 ± 152.39	*p* < 0.05	282.09 ± 53.98	n.s
3	RANTES	22.74 ± 3.48	9.14 ± 2.35	*p* < 0.01	2.97 ± 1.81	*p* < 0.0001
4	GM-CSF	1.72 ± 0.81	18.5 ± 4.01	*p* < 0.01	0.45 ± 0	n.s
5	LIF	3.16 ± 0.33	16.14 ± 1.82	*p* < 0.001	4.76 ± 0.42	n.s
6	MCP-1	23 ± 5.98	97.03 ± 20.24	*p* < 0.001	15.5 ± 1.63	n.s
7	IL-2	30.45 ± 5.16	16.05 ± 4.4	n.s	5.36 ± 3.96	*p* < 0.05
8	IL-4	1.73 ± 0.35	0.93 ± 0.19	n.s	0.27 ± 0.06	*p* < 0.05
9	IL-5	1.14 ± 0.19	0.64 ± 0.23	n.s	0.4 ± 0.08	*p* < 0.05
10	IL-9	123.77 ± 17.15	67.88 ± 19.32	n.s	24.11 ± 15.9	*p* < 0.05
11	IL-13	6.2 ± 1.1	3.58 ± 0.7	n.s	2.04 ± 0.27	*p* < 0.05
12	MIP-1β	49 ± 7.27	45.38 ± 9.36	n.s	17.61 ± 2.29	*p* < 0.05
13	MDC	14.12 ± 1.38	6.92 ± 2.54	n.s	5.29 ± 0.97	*p* < 0.05
14	Eotaxin	324.26 ± 15.66	346.38 ± 101.8	n.s	34.57 ± 21.06	n.s
15	G-CSF	2.02 ± 0.22	4.89 ± 1.69	n.s	2.92 ± 0.5	n.s
16	IFNγ	4.55 ± 0.53	3.75 ± 1.14	n.s	4.18 ± 0.8	n.s
17	IL-1α	121.84 ± 13.94	96.47 ± 12.64	n.s	81.4 ± 6.21	n.s
18	IL-1β	15.91 ± 2.34	11.06 ± 2.21	n.s	8.28 ± 0.27	n.s
19	IL-3	b.d	b.d	n.s	b.d	n.s
20	IL-6	3.6 ± 0.14	5.83 ± 1.74	n.s	4.86 ± 0.58	n.s
21	IL-7	10.41 ± 2.52	6.26 ± 1.71	n.s	3.13 ± 1.23	n.s
22	IL-10	47.89 ± 2.93	27.25 ± 6.85	n.s	26.5 ± 2.31	n.s
23	IL-12p40	11.11 ± 2.34	5.21 ± 1.52	n.s	10.88 ± 2.03	n.s
24	IL-12p70	b.d	b.d	n.s	b.d	n.s
25	IL-15	47.12 ± 10.27	22.24 ± 5.35	n.s	16.72 ± 4.65	n.s
26	IL-17	16.82 ± 4.53	16.67 ± 4.94	n.s	2.35 ± 1.26	n.s
27	IP-10	37.03 ± 4.84	50.28 ± 14.45	n.s	5.37 ± 3.34	n.s
28	KC	41.05 ± 3.5	43.7 ± 14.93	n.s	16.55 ± 3.56	n.s
29	MIG	715.16 ± 171.53	1302.87 ± 524.66	n.s	106.75 ± 74.75	n.s
30	MIP-1α	25.79 ± 4.29	45.27 ± 6.09	n.s	25.08 ± 3.46	n.s
31	MIP-2	215.85 ± 25.28	175.98 ± 25.08	n.s	160.72 ± 13.27	n.s
32	TNFα	2.95 ± 0.86	4.88 ± 1.46	n.s	0.88 ± 0.3	n.s
33	VEGF	35.16 ± 7.69	17.33 ± 6.47	n.s	38.02 ± 4.49	n.s
34	6Ckine/Exodus 2	1246.48 ± 159.41	4785.37 ± 3818.81	n.s	821.86 ± 239.75	n.s
35	EPO	93.9 ± 35.11	37.71 ± 21.7	n.s	147.72 ± 34.56	n.s
36	Fractalkine	938.65 ± 115.42	520.75 ± 171.04	n.s	529.81 ± 103.02	n.s
37	IFNβ-1	252.83 ± 44.12	92.82 ± 19.95	n.s	318.49 ± 72.79	n.s
38	IL-11	631.25 ± 124.99	193.75 ± 49.54	n.s	785.42 ± 195.99	n.s
39	IL-16	640.47 ± 93.76	387.31 ± 118.71	n.s	334.19 ± 33.16	n.s
40	IL-20	39.94 ± 4.59	21.38 ± 2.34	n.s	38.9 ± 7.28	n.s
41	MCP-5	126.93 ± 14.83	447.78 ± 156.78	n.s	204 ± 35.6	n.s
42	MIP-3α	5.11 ± 0.94	2.39 ± 0.31	n.s	9.17 ± 2.05	n.s
43	MIP-3β	76.22 ± 10.04	90.66 ± 19.28	n.s	84.96 ± 9.44	n.s
44	TARC	21.57 ± 2.48	59.78 ± 23.06	n.s	8.88 ± 0.63	n.s

## Data Availability

The data presented in this study are available from the authors on reasonable request.
